# Foot Sole Temperature Correlates with Ankle–Brachial Index, Pulse Wave Velocity, and Arterial Age in Diabetic Patients Without Diagnosis of Peripheral Arterial Disease

**DOI:** 10.3390/jcm13216383

**Published:** 2024-10-25

**Authors:** Blanca Estela Ríos-González, Liliana López-Barragán, Ana Miriam Saldaña-Cruz, Sergio Gabriel Gallardo-Moya, Aniel Jessica Leticia Brambila-Tapia, Carlos Eduardo Soto-Ramirez, Elida Berenice Garcia-Calvario

**Affiliations:** 1 Unidad de Medicina Familiar No. 92, Instituto Mexicano del Seguro Social, Guadalajara 44990, Jalisco, Mexico; 2 Departamento de Fisiología, Centro Universitario de Ciencias de la Salud (CUCS), Universidad de Guadalajara, Guadalajara 44340, Jalisco, Mexico; ana.saldanac@academicos.udg.mx (A.M.S.-C.); sergio.gallardo@alumnos.udg.mx (S.G.G.-M.); 3 Departamento de Psicología Básica, Centro Universitario de Ciencias de la Salud (CUCS), Universidad de Guadalajara, Guadalajara 44340, Jalisco, Mexico; aniel.brambila@academicos.udg.mx; 4 Hospital General de Zona No. 14, Instituto Mexicano del Seguro Social, Guadalajara 44860, Jalisco, Mexico; 5 Unidad de Medicina Familiar No. 88, Instituto Mexicano del Seguro Social, Guadalajara 44987, Jalisco, Mexico

**Keywords:** ankle–brachial index, pulse wave velocity, arterial age, thermography, diabetes

## Abstract

**Background/Objectives**: Some vascular alterations such as peripheral arterial disease (PAD) or arterial stiffness can alter perfusion of the limbs, so we wondered if this is reflected in the temperature of the soles of the feet of diabetic patients who did not have a diagnosis of peripheral arterial disease. Foot sole temperature was correlated with the ankle–brachial index (ABI), carotid—femoral pulse wave velocity (cfPWV), brachial–ankle pulse wave velocity (baPWV), and arterial age. **Methods:** A total of 175 patients with type 2 diabetes mellitus, without a previous diagnosis of PAD, were recruited. Comorbidities, anthropometry, biochemical analysis results, temperature, ABI, cfPWV, baPWV, and arterial age were recorded. Forty-two temperature records were obtained from the sole of the foot with an FLIR T865 thermal imaging camera. ABI, cfPWV, baPWV, and arterial age were obtained using plethysmographic and oscillometric methods. Statistical analysis was performed with SPSS v.29.0 (correlations and multiple linear regression models). **Results:** All temperature points analyzed correlated negatively with ABI (*p* < 0.001) and rho values ranged from −0.168 to −0.210. Likewise, cfPWV, baPWV, and arterial age had similar results, since most temperature records showed low rho values and a negative correlation with these parameters. Four models were developed to explain the variables of interest. Temperature was involved in all of them. The temperature of the first toe was included in the prediction of cfPWV, baPWV, and arterial age. **Conclusions:** There is an inversely proportional relationship between temperature and ABI, cfPWV, baPWV, and arterial age in diabetic patients without a previous diagnosis of arterial disease. Temperature can be a predictor of these hemodynamic variables.

## 1. Introduction

There are more than 200 million people with peripheral arterial disease (PAD) globally. In diabetic patients, the prevalence is 25.6% [1], and even when it causes manifestations such as intermittent claudication, hair loss, and pain in the lower extremities, among others, this condition is underdiagnosed because it can be asymptomatic in many patients [2]. Therefore, there is a need to search for new diagnostic or screening methods to carry out an early diagnosis to avoid the morbidity and mortality associated with PAD. One of the quickest and most economical methods to diagnose peripheral arterial disease is the ankle–brachial index (ABI), which is considered diagnostic when indices are obtained with values < 0.9 (the lower the value, the greater the severity of PAD) or >1.4 (artery calcification) [3,4]. This disease is characterized by the formation of atheromatous plaques in arteries in the lower extremities; this directly affects perfusion and the temperature of the affected extremity.

Other processes in the vasculature can affect perfusion, such as arterial stiffness, which can be measured through pulse wave velocity (PWV); this is a measure of the speed of arterial pressure waves traveling along the aorta and large arteries, and it is usually calculated by dividing the distance between two points on the arteries being recorded by the pressure wave transit time. Therefore, there are different variants, such as carotid–femoral pulse wave velocity (cfPWV) and brachial–ankle pulse wave velocity (baPWV), with the first being the gold standard [5]. There are devices that, through measurement of the pressure in the four limbs, can calculate ABI and pulse wave velocity and can also obtain an arterial age value (through the application of algorithms). Arterial age can be compared with chronological age and provide us with data on vascular damage [6].

Thermography is a non-invasive and precise tool that could show these possible temperature changes, especially in the plantar area in diabetic patients, which is the most susceptible to ulceration, complications, and amputation [7]. Therefore, it is necessary to search for new methodologies to identify flow alterations in the lower extremities that can predict or complement other tools such as the ABI, PWV, and arterial age. The aim of the study was to correlate temperature with ABI, PWV, and arterial age in the soles of the feet of diabetic patients without a diagnosis of PAD and to perform regression models for ABI, PWV, and arterial age in these patients.

## 2. Materials and Methods

Subjects: The study group was formed of 175 patients with type 2 diabetes mellitus without a previous diagnosis of peripheral arterial disease (PAD). The selection was based on reviewing the primary and secondary healthcare clinical records from the last 5 years (350 feet analyzed). If any patient had a PAD diagnosis, or an imaging study that corroborated PAD in their clinical record, they were not included. Although arteriography is the gold standard for PAD, it is not routinely used because it is very invasive and involves a risk due to radiation [8]. This is why it is reserved only for patients who require surgical treatment. The patients were recruited over six months (January–June 2023). The sample size was calculated using the formula for bivariate correlations, with a precision of 0.05, confidence of 95%, and a statical power of 80%, for a total of 67 patients [9]. Exclusion criteria included pregnant women, patients with type 1 diabetes, limb amputees, and patients with local inflammation processes such as an infection or phlebitis. Patients were recruited from a family medical unit at the Mexican Social Security Institute (primary care) in Jalisco, Mexico. They were informed about the objectives of the study and agreed to sign an informed consent form. The protocol was approved by an ethics committee (COPEPRIS 17 CI 14 039 058, Registro CONBIOETICA 14-CEI-004-20211201) and the Helsinki principles were respected.

The following variables were recorded for all participants:

(a) Comorbidities and smoking index (number of cigarettes smoked per day) × (number of years of smoking)/20. Smoking risk was classified as follows: <10 null, 10–20 moderate, 21–40 intense, >41 high.

(b) Biochemical variables: blood count (hemoglobin, hematocrit, platelets, leukocytes), glucose, creatinine, glomerular filtration (CKD-EPI equation), uric acid, total cholesterol, low-density lipoprotein (LDL), high-density lipoproteins (HDL), very low-density lipoprotein (VLDL), triglycerides, glycosylated hemoglobin A1c and average glucose (28.7 × hemoglobin A1c −46.7).

(c) Anthropometric variables: weight (kg), height (m), body mass index (BMI), systolic and diastolic blood pressure (mmHg) (SBP and DBP).

(d) Hemodynamic variables: The ABI, cfPWV, baPWV, and arterial age were obtained using MESI-ABI equipment (Leskoškova cesta 11, 1000 Ljubljana, Slovenia), an improved oscillometric method, and plethysmography with the PADsense algorithm. Arterial age is calculated automatically by the MESI device and the pulse wave velocity application, as reported by Mattace-Raso et al. (2010) [10].

For proper measurement, the patient remained in a supine position for at least 5 min before measurement. Peripheral arterial disease with an abnormal ABI was classified as mild 0.7–0.9, moderate 0.5–0.69 or severe < 0.49.

Arterial stiffness was diagnosed if the cfPWV values were >2 standard deviations, according to the age ranges reported by Baier et al. (2018) [11]. Additionally, we evaluated how many patients had abnormal baPWV values, using the same reference (Baier et al., 2018 [11]).

**Figure 1:**
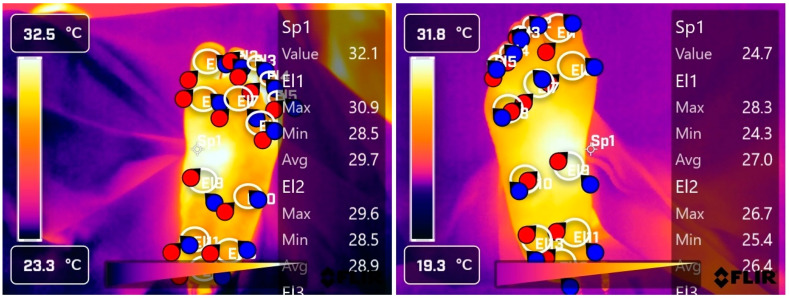
Areas of the sole of the foot were analyzed with a thermal imaging camera. The white and yellow areas (warm colors) represent higher temperatures, while the purple and blue regions (cold colors) represent areas with lower temperatures. R: right foot; L: left foot; T: temperature; EI1: area T1 (first toe); EI2: area T2 (second toe); EI3: area T3 (third toe); EI4 area T4 (fourth toe); EI5 area T5 (fifth toe); EI6: area T6 (first metatarsal)); EI7: area T7 (third metatarsal); EI8: area T8 (fifth metatarsal); EI9: area T9 (internal arch); EI10: area T10 (external arch); EI11: area T11 (inner heel); EI12: area T12 (central heel); EI13: area T13 (external heel).

(e) Temperature (T): The temperatures of 13 points in the plantar area were measured: T1 (first toe), T2 (second toe), T3 (third toe), T4 (fourth toe), T5 (fifth toe), T6 (first metatarsal), T7 (third metatarsal), T8 (fifth metatarsal), T9 (internal arch), T10 (external arch), T11 (inner heel), T12 (central heel), and T13 (external heel) (Figure 1). The maximum, minimum, and average temperature at each point was recorded, as well as the average maximum (1–13 points), average minimum (1–13 points), and the overall average (1–13 points). In total, 42 temperature records were obtained on each foot. We used an FLIR T865 thermal imaging camera, with a resolution of 640 × 480 pixels, a temperature range from −40 to 2000 °C and a precision of ±1 °C. Images were taken in an area heated to 21 degrees Celsius and humidity was measured by a hygrometer. Emissivity was 0.98, for human skin. Participants were asked not to apply products such as cream, powder, or antiperspirants that could alter the images. Patients remained in the heated area for at least 15 min, with their feet uncovered, then were placed on an examination bed in a supine position for at least 5 min. The camera was placed on a tripod at a distance of 80 cm from the patients’ feet and images of both feet were subsequently captured. The images were analyzed using the FLIR Thermal program, obtaining the average, minimum, and maximum temperatures of the 13 analyzed points.

Statistical analysis was carried out using the SPSS v.29.0 program. The Kolmogorov–Smirnov normality test was performed on the variables of temperature, ABI, PWV, and vascular age. Subsequently, Spearman correlation tests were performed to determine bivariate correlations between the hemodynamic variables and temperature (considering the distribution was non-parametric), and, finally, multiple linear regressions were performed with the step-wise method for hemodynamic variables and temperature.

## 3. Results

A total of 175 diabetic patients were analyzed; 62.9% were women and 37.1% men. The mean chronological age was 61.05 ± 11.96. Based on the BMI recommendations proposed by the World Health Organization, 16.6% had a normal BMI and 33.7% were overweight or some degree of obese (OI 24.6%, OII 17.7%, and OIII 7.4%). The following comorbidities were found: Hypertension was the most frequent comorbidity, exhibited in 113 individuals (64.57%), followed by dyslipidemia in 22 (12.57%), and third was heart disease in 8 (4.57%). Likewise, other diseases with frequencies less than 2% were recorded, such as hypothyroidism in three people (1.71%), chronic obstructive pulmonary disease in two (1.14%), vitiligo in two (1.14%), asthma in one (0.57%), Parkinson’s in one (0.57%), a cerebral stroke in one (0.57%), hyperprolactinemia in one (0.57%), and cysticercosis in one (0.57%).

Only 25 patients (14.3%) had a glomerular filtration rate of less than 60 mL/min (G3a, 18 (10.3%); G3b, 6 (3.4%); G4, 1 (0.6%)) and no one had kidney failure (G5). Smoking was present in 71 (40.6%), of these patients; the risk according to the smoking index was null in 44 patients (62.0%), moderate in 13 (18.3%), intense in 10 (14.1%), and high in 4 (5.6%).

The number of patients within the control goal limits outlined by the America Diabetes Association 2023 were as follows: 81 (46.3%) for systolic pressure (<130 mmHg), 104 (59.4%) for diastolic pressure (<80 mmHg), 45 (25.7%) for LDL (<70 mg/dL), and 98 (56.0%) for HbA1c (<7%).

The means and standard deviations for each of the biochemical, anthropometric, and hemodynamic variables were calculated and are shown in Table 1. Some averages did not meet the control goals such as BMI, which is in the obesity range, SBP, HbA1c, triglycerides, and LDL.

Although all participants denied diagnosis of peripheral arterial disease, four patients (2.3%) had an abnormal ABI value, two (1.1%) had a mild value, one (0.6%) a moderate value, and one (0.6%) a severe value. No one had an ABI > 1.4. No one had arterial stiffness based on cfPWV. However, baPWV values were abnormal for up to 72 foot measurements (20.6%). The numbers and percentages of patients according to age were as follows: 18–29 years old, four patients (1.2%); 30–39, thirteen (3.7%); 40–49, twenty-six (7.4%); 50–59, fifteen (4.3%); 60–69, thirteen (3.7%); and >70, one (0.03%). Two baPWV values were missing.

The mean chronological age was 61.05 ± 11.96, while arterial age was lower, with a mean of 54.15 + 14.19.

### 3.1. Temperature Correlates with ABI, PWV, and Arterial Age

All correlations of temperature (42 records) with ABI were statistically significant and negative; that is, the lower the temperature, the higher the ankle–brachial index. However, the correlation coefficients were low, since the rho values had a range of −0.168 to −0.210 (Table 2).

The variables cfPWV, baPWV, and arterial age had similar results—32 of the 42 temperature records showed statistical significance (one less for baPWV), with negative coefficients and low rho values. Therefore, the lower the temperature, the higher the cfPWV, baPWV, and arterial age (Table 2).

### 3.2. Regression Models of Temperature with ABI, PWV, and Arterial Age

Four models were established to explain the four variables of interest, and temperature was involved in all of them. The intercept, slope coefficient, R, R2, and significance are shown in Table 3.

Temperature played an important role, since of the seven variables involved in the ABI model, three were temperatures (42.9%), while in the cfPWV model, four of the eleven variables involved were temperatures (36.4%). For baPWV, two of the seven variables were temperatures (28.6%) and, finally, for arterial age, four of the twelve variables were temperatures (33.3%).

The first model is estimated to explain up to 26.2% of ABI with SBP, T6min (minimum temperature of the first metacarpal), leukocytes, glucose, smoking index, T4max (maximum temperature of the fourth toe), and T5max (maximum temperature of the fifth toe).

Up to 55.2% of cfPWV can be estimated using the following variables: chronological age, BPD, T1max (maximum temperature of the first toe), HbA1c, BMI, SBP, T9max (maximum temperature of the internal arch), gender (male), T11max (maximum temperature of the inner part of the heel), T1Mean (mean temperature of the first toe) and triglycerides.

The third model obtained the highest R2 of the four models, explaining up to 56.5% of baPWV with chronological age, SBP, HbA1c, T1max, BMI, T3min (minimum temperature of the third toe), and DBP.

The final model explains arterial age, which can be related to chronological age, DBP, T1max, HbA1c, BMI, SBP, T1Mean, T9max, T11max, gender, triglycerides, and uric acid by up to 56%.

## 4. Discussion

As in this study, De Carvalho Abreu et al. (2021b) [7] sought to correlate ABI with temperature, but their results differed from ours since they found a positive correlation between ABI and temperature. However, we must highlight differences in studies, such as sample size, which was smaller in their study compared to ours (72 vs. 350 feet), and the study group (patients with a diagnosis of PAD vs. diabetic patients without a previous diagnosis of PAD). What they reported can be explained by the fact that patients diagnosed with PAD present a decrease in the vascular lumen, which would reduce blood flow and therefore lower the temperature [12], while in diabetic patients without PAD, when ABI decreases in the initial stages (without reaching the diagnostic cut-off point of <0.9), the increase in temperature could be secondary to an inflammatory process that accompanies the formation of atheroma plaques [13], or by the fact that flow initially does not decrease until the atheromatous plaque exceeds 40% obstruction, and before reaching this stage there is a compensatory arterial enlargement [14]. Another possible explanation for our results could be that our group was made up of diabetic patients following the guidelines on the diagnosis, prognosis, and management of PAD in patients with foot ulcers and diabetes [15], it has not been possible to define an ideal diagnostic method for diabetic patients, since all tools have limitations. Therefore, in future studies, another tool or a combination could be useful, like the toe–brachial index (TBI) or triphasic pedal Doppler waveforms.

With respect to the arterial age being lower than the chronological one, this apparently contradictory result can be explained by the selection criteria excluding patients who had not had any vascular symptoms that warranted referral to angiology or imaging studies that suggested PAD, in the last five years. It is likely that, if we included all patients with diabetes, the arterial age would be higher. Another possible explanation is that the arterial age was calculated using an oscillometry device (MESI) based on extrapolating the PWV with the values reported in the healthy population at different chronological ages, and it does not consider other factors or comorbidities.

On the other hand, to our knowledge, there are no similar studies that have correlated variables like PWV, arterial age, and thermography. The only study previously attempted was a model that used the temperature of skin surfaces and cfPWV, baPWV, and ABI, among other hemodynamic variables, to try to predict an increase in blood pressure with a computer-aided diagnostics model; the highest accuracy of 89% was achieved by combining variables like mean skin surface temperature measured from static and dynamic infrared thermograms and PWV variables [16].

Our results on the negative correlations of temperature with cfPWV, baPWV, and arterial age, could be due to a decrease in elastic fibers, which compromises vasodilation and thus blood flow; as flow decreases, temperature decreases [17]. Additionally, our patients have diabetes mellitus, which promotes the production of advanced glycation end products, which are a risk factor for arterial stiffness [18].

Regarding the regression models, how could we explain the higher participation of certain temperature points compared to others? To the best of our knowledge, there are no similar studies to compare with ours, but it is worth highlighting that, of the 13 points analyzed on the sole, the most constant site (being present in the regression models for cfPWV, baPWV, and arterial age) was that of the first toe (T1), which is irrigated by the posterior tibial artery. Temperatures 4, 5, and 6 were involved in the ABI model. Finally, points T9 and T11 are also included in the cfPWV and arterial age models, and their perfusion occurs through the median and lateral calcaneal arteries, respectively [19].

The first limitation of our study is that the diagnosis was based on the patients’ clinical history; however, imaging studies like angiography, (which is the gold standard), are only performed when the patient requires surgical intervention, due to the risks of this type of study [8]. Another limitation of our study is that only ABI was used. As mentioned in the guidelines on the diagnosis, prognosis, and management of PAD in patients with foot ulcers and diabetes [14], there is no ideal method. However, when there is calcification (which is very frequent in patients with DM2) [20] and kidney failure, TBI becomes the most appropriate methodology because hyalinization of the vessel intima leads to a falsely high ABI (above 1.4–1.5), which makes the diagnosis and treatment of PAD difficult, especially in small vessel atherosclerosis in the tibial, peroneal, or dorsal arteries of the foot and metatarsals. In these patients, the toe–brachial index (TBI) is evaluated using ultrasound, with a small cuff placed on the big toe. A TBI value of <0.7 is diagnostic of PAD [21].

Another limitation was not including tests that assess PAD symptoms, such as intermittent claudication. Therefore, if future studies can use a combination of both diagnostic tools and symptom assessment, they could be more precise. Another limitation of our study was that we did not specifically record which hypoglycemic agents our study group ingested. This would be important to include in future research, due to possible interactions with the ABI, PWV, and arterial age.

Future perspectives: We could screen patients with an abnormal ABI and observe what happens with their temperature (which has already been reported but with reduced sample sizes). Another interesting option in terms of the clinical thermography of diabetics would be to follow up on those individuals who showed an increase in the temperature of the sole and to find out how many developed PAD. It should be noted that, although 44% of participants had Hb1Ac values > 7, any damage related to an altered ABI, pWV, and vascular age was minimal; this can be explained by the non-inclusion criteria, but it is likely that, when these patients were followed up, several would have developed vascular dysfunction, as has been previously reported in the literature [22]. This would determine whether or not thermography has potential as a predictive tool.

## 5. Conclusions

There is an inversely proportional relationship between temperature with ABI, cfPWV, baPWV, and arterial age in diabetic patients without a previous diagnosis of PAD. Temperature can be related to these hemodynamic variables.

## Figures and Tables

**Table 1:** Means and standard deviations of biochemical, anthropometrical, and hemodynamic variables.

Variables	Mean	Standard Deviation
BMI	30.81	6.19
Smoking index	4.82	11.13
SBP (mm/Hg)	134.58	19.32
DBP (mm/Hg)	79.17	8.95
ABI	1.16	0.13
cfPWV (m/s)	8.50	1.70
baPWV (m/s)	13.46	2.89
Arterial age (years)	54.15	14.19
Glycosylated hemoglobin (%)	7.39	1.97
Glucose (mg/dL)	123.06	67.27
Creatinine (mg/dL)	0.94	0.26
Uric acid (mg/dL)	4.24	1.21
Total cholesterol (mg/dL)	177.96	144.28
Triglycerides (mg/dL)	163.90	158.41
HDL (mg/dL)	42.63	13.01
LDL (mg/dL)	93.54	36.21
VLDL (mg/dL)	30.96	16.25

BMI: body mass index; SBP: systolic blood pressure; DBP: diastolic blood pressure; cfPWV: carotid–femoral pulse wave velocity; baPWV: brachial–ankle pulse wave velocity; HDL: high-density lipoprotein; LDL: low-density lipoprotein; VLDL: very low-density lipoprotein.

**Table 2:** Spearman correlation coefficients (rho) for temperature and ankle–brachial index, pulse wave velocity, and arterial age.

Temperature °C (Mean ± Standard Deviation)		ABI	cfPWV	baPWV	Arterial Age
T1 (26.797 ± 3.089)	Max	−0.199 **	−0.142 **	−0.127 *	−0.142 **
	Min	−0.170 **	−0.097	−0.083	−0.095
	Mean	−0.181 **	−0.106 *	−0.091	−0.106 *
T2 (25.797 ± 3.055)	Max	−0.202 **	−0.104	−0.082	−0.100
	Min	−0.193 **	−0.074	−0.056	−0.076
	Mean	−0.209 **	−0.084	−0.064	−0.081
T3 (25.237 ± 2.992)	Max	−0.184 **	−0.123 *	−0.107 *	−0.119 *
	Min	−0.199 **	−0.072	−0.059	−0.068
	Mean	−0.204 **	−0.076	−0.062	−0.072
T4 (25.472 ± 2.992)	Max	−0.168 **	−0.119 *	−0.108 *	−0.113 *
	Min	−0.187 **	−0.062	−0.057	−0.061
	Mean	−0.189 **	−0.083	−0.075	−0.078
T5 (25.715 ± 2.952)	Max	−0.173 **	−0.132 *	−0.125 *	−0.126 *
	Min	−0.176 **	−0.097	−0.092	−0.096
	Mean	−0.181 **	−0.099	−0.092	−0.094
T6 (26.844 ± 2.583)	Max	−0.182 **	−0.161 **	−0.148 **	−0.163 **
	Min	−0.175 **	−0.137 *	−0.125*	−0.140 **
	Mean	−0.173 **	−0.148 **	−0.135*	−0.151 **
T7 (27.079 ± 2.687)	Max	−0.195 **	−0.149 **	−0.140 **	−0.145 **
	Min	−0.187 **	−0.141 **	−0.134*	−0.143 **
	Mean	−0.187 **	−0.148 **	−0.141 **	−0.149 **
T8 (26.844 ± 2.583)	Max	−0.173 **	−0.155 **	−0.149 **	−0.150 **
	Min	−0.168 **	−0.139 **	−0.136 *	−0.135 *
	Mean	−0.175 **	−0.144 **	−0.140 **	−0.142 **
T9 (28.362 ± 2.279)	Max	−0.217 **	−0.143 **	−0.132 *	−0.139 **
	Min	−0.177 **	−0.150 **	−0.146 **	−0.147 **
	Mean	−0.210 **	−0.139 **	−0.131 *	−0.136 *
T10 (27.073 ± 2.320)	Max	−0.189 **	−0.160 **	−0.154 **	−0.158 **
	Min	−0.181 **	−0.152 **	−0.139 **	−0.149 **
	Mean	−0.184 **	−0.157 **	−0.147 **	−0.154 **
T11 (26.717 ± 2.491)	Max	−0.195 **	−0.150 **	−0.147 **	−0.144 **
	Min	−0.182 **	−0.125 *	−0.121 *	−0.119 *
	Mean	−0.198 **	−0.127 *	−0.123 *	−0.121 *
T12 (26.101 ± 2.501)	Max	−0.194 **	−0.131 *	−0.130 *	−0.123 *
	Min	−0.182 **	−0.116 *	−0.112 *	−0.107 *
	Mean	−0.193 **	−0.117 *	−0.114 *	−0.110 *
T13 (26.695 ± 2.466)	Max	−0.186 **	−0.149 **	−0.143 **	−0.143 **
	Min	−0.196 **	−0.134 *	−0.121 *	−0.126 *
	Mean	−0.189 **	−0.135 *	−0.129 *	−0.128 *
Mean T1–T13 (26.362 ± 2.623)	Max	−0.197 **	−0.146 **	−0.137 *	−0.142 **
	Min	−0.197 **	−0.125 *	−0.114 *	−0.121 *
	Mean	−0.198 **	−0.126 *	−0.116 *	−0.123 *

* *p* < 0.05, ** <0.001, T: temperature; Max: maximum; Min: minimum; cfPWV: carotid–femoral pulse wave velocity; baPWV: brachial–ankle pulse wave velocity; ABI: ankle-brachial index.

**Table 3:** Regression models involving temperature for ABI, PWV, and arterial age.

Formula: (Y = *β0 + β1X1 + β2X2 + ….ε*).						
Dependent Variable (Y)	Intercept (*β0*)	Independent Variable (X)	Slope Coefficient (β)	R	R^2^	Significance
ABI	1.904	SBP	−0.413	0.511	0.262	<0.001
		T6min	−0.427			
		Leukocytes	−0.219			
		Mean Glucosa	0.170			
		Smoking index	−0.145			
		T4max	0.698			
		T5max	−0.462			
cfPWV	0.334	Age	0.438	0.728	0.560	<0.001
		DBP	0.204			
		T1max	−1.101			
		HbA1c	0.196			
		BMI	−0.125			
		SBP	0.194			
		T9max	0.466			
		Male Gender	0.121			
		T11max	−0.383			
		T1Mean	0.843			
		Triglycerides	−0.089			
baPWV	0.336	Age	0.549	0.751	0.565	<0.001
		SBP	0.252			
		HbA1c	0.180			
		T1max	−0.369			
		BMI	−0.104			
		T3min	0.223			
		DBP	0.111			
Arterial age	−24.876	Age	0.479	0.748	0.560	<0.001
		DBP	0.258			
		T1max	−1.186			
		HbA1c	0.209			
		BMI	−0.110			
		SBP	0.150			
		T1Mean	0.951			
		T9max	0.436			
		T1max	−0.364			
		Male Gender	0.069			
		Triglycerides	−0.127			
		Uric acid	0.097			

ABI: ankle–brachial index; SBP: systolic blood pressure; DBP: diastolic blood pressure; BMI: Body mass index; T: temperature; HbA1c: glycosylated hemoglobin A1c; max: maximum; min: minimum.

## Data Availability

The original contributions presented in the study are included in the article, further inquiries can be directed to the corresponding authors.
